# Resting-State EEG Signature of Early Consciousness Recovery in Comatose Traumatic Brain Injury Patients

**DOI:** 10.21203/rs.3.rs-3895330/v1

**Published:** 2024-01-31

**Authors:** Ayham Alkhachroum, Emilia Flo, Brian Manolovitz, Holly Marie Stradecki-Cohan, Berje Shammassian, Danielle Bass, Gabriela Aklepi, Esther Monexe, Pardis Ghamasaee, Evie Sobczak, Daniel Samano, Ana Bolaños Saavedra, Nina Massad, Mohan Kottapally, Amedeo Merenda, Joacir Graciolli Cordeiro, Jonathan Jagid, Andres M. Kanner, Tatjana Rundek, Kristine O'Phelan, Jan Claassen, Jacobo Sitt

## Abstract

Background Resting-state electroencephalogram (rsEEG) is usually obtained to assess seizures in comatose patients with traumatic brain injury (TBI) patients. We aim to investigate rsEEG measures and their prediction of early recovery of consciousness in comatose TBI patients. Methods This is a retrospective study of comatose TBI patients who were admitted to a level-1 trauma center (10/2013-1/2022). Demographics, basic clinical data, imaging characteristics, and EEG data were collected. We calculated using 10-minute rsEEGs: power spectral density (PSD), permutation entropy (PE – complexity measure), weighted symbolic-mutual-information (wSMI – global information sharing measure), Kolmogorov complexity (Kolcom – complexity measure), and heart-evoked potentials (HEP - the averaged EEG signal relative to the corresponding QRS complex on electrocardiogram). We evaluated the prediction of consciousness recovery before hospital discharge using clinical, imaging, rsEEG data via Support Vector Machine with a linear kernel (SVM). Results We studied 113 (out of 134, 84%) patients with rsEEGs. A total of 73 (65%) patients recovered consciousness before discharge. Patients who recovered consciousness were younger (40 vs. 50, p .01). Patients who recovered consciousness had higher Kolcom (U = 1688, p = 0.01,), increased beta power (U = 1652 p = 0.003), with higher variability across channels ( U = 1534, p = 0.034), and epochs (U = 1711, p = 0.004), lower delta power (U = 981, p = 0.04) and showed higher connectivity across time and channels as measured by wSMI in the theta band (U = 1636, p = .026, U = 1639, p = 0.024) than those who didn’t recover. The ROC-AUC improved from 0.66 (using age, motor response, pupils’ reactivity, and CT Marshall classification) to 0.69 (p < 0.001) when adding rsEEG measures. Conclusion We describe the rsEEG EEG signature in recovery of consciousness prior to discharge in comatose TBI patients. Resting-state EEG measures improved prediction beyond the clinical and imaging data.

## Introduction

Traumatic brain injury (TBI) is a major cause of death and disability.[1] One quarter of severe TBI patients die, and over 60% of these deaths are influenced by the decision to withdraw life-sustaining therapies.[2]

Clinicians and patients' surrogates are often faced with uncertainty when determining the prognosis for recovery of consciousness in comatose patients.[3,4] The current prognostication models use age, basic neurological assessments, and radiographic findings as predictors.[5–7] Agreement between observed and predicted probabilities is poor, highlighting the inaccuracy of these models. Both mortality and unfavorable outcomes are generally lower than predicted.[5,7,8]

Electroencephalogram (EEG) is recorded as a standard of care in patients with severe TBI to identify seizures. The underlying neurophysiologic brain activity can be analyzed using the EEG signal.[9] More recently, brain activation to motor commands (task-based EEG) identified patients with cognitive motor dissociation (CMD) early after acute brain injury.[10] These patients were more likely to be independent at 12-month follow-up and had a faster recovery.[10,11] However, evaluation of CMD is limited to a few centers as it requires expertise in obtaining and analyzing the data. Alternatively, resting-State EEG (rsEEG) is widely available and with relatively low expense.[9] Analyses of rsEEG exploring the signal power, amplitude, complexity, and inter-signal relationships may improve the ability to accurately prognosticate recovery after brain injury.[9] Despite the current guidelines recommending the use of EEG for the evaluation of consciousness after brain injury, studies on using rsEEG for prognostication in the intensive care unit (ICU) are limited.[12,13]

In this study, we aim to evaluate the utility of rsEEG quantitative measures for the prediction of recovery of consciousness prior to hospital discharge in comatose TBI patients. We hypothesized that rsEEG measures are different between the two groups and would improve prediction of consciousness recovery, beyond the basic demographics, clinical and imaging data.

## Methods

This is a retrospective single center study performed between October 2013 and January 2022 at Jackson Memorial Hospital/Ryder Trauma Center. The study was approved by the University of Miami IRB and the Jackson Memorial Research office. Data were collected in *REDcap* (a secure web application for managing databases, approved by the University IRB).[14,15] For data collection, we used the National Institute of Neurological Disorders and Stroke Common Data Elements for TBI.[16]

**Subjects.** We identified retrospectively 134 TBI patients (18 years and older) that were admitted to the intensive care units at our institution and were monitored with EEG during their hospital stay. All patients were comatose on admission, defined as eyes closed with the inability to follow commands as evaluated by a neurologist or a neurosurgeon.

**Clinical and Imaging Data Collection.** We collected the patients' basic demographics (age, sex, race/ethnicity), mechanism of injury, type of injury (subdural hematoma (SDH), epidural hematoma (EDH), subarachnoid hemorrhage (SAH), contusion, and diffuse axonal injury (DAI)), Glasgow Coma Scale on admission (GCS), Charlson Comorbidity Index (CCI) on admission, pupils' reactivity, surgical treatments (neurological, tracheostomy, gastrostomy (PEG)), and seizures. Type of injury and Marshall

CT classification was adjudicated by a neurosurgeon.[17] We collected the cumulative received doses over the 2 previous half-lives of midazolam (24 hours), propofol (2 hours), fentanyl (8 hours), ketamine (6 hours), and dexmedetomidine (4 hours). Sedation levels were classified as follows: “none” if no sedation; “minimal” if only pushes of sedatives; “low” for continuously administrated doses of midazolam (≤ 0.15 mg/kg/h), propofol (≤ 4 mg/kg/h), fentanyl (≤ 2 µg/kg/h), or dexmedetomidine (any dose); “moderate” for continuously administrated doses of midazolam (> 0.15 mg/kg/h), propofol (> 4 mg/kg/h), fentanyl (> 2 µg/kg/h), or ketamine (any dose); and “deep” for barbiturate infusions.

We collected the following outcomes: hospital and ICU length of stay, Glasgow Outcome Scale-Extended (GOS-E) on discharge, mortality, withdrawal of life-sustaining therapies and discharge disposition. GOS-E is an eight category scale and is the most commonly used scale for global outcomes after TBI: (1) dead, (2) vegetative state (patient has no clinical evidence of awareness), (3) lower severe disability (patient is dependent and cannot be left alone for more than 8 hours at home), (4) upper severe disability (patient is dependent and can be left alone for more than 8 hours at home), (5) lower moderate disability (patient is independent at home but not able to return to work), (6) upper moderate disability (patient is independent at home and able to return to work with special arrangements), (7) lower good recovery (patient is able to resume normal life with the capacity to work with disabling neurological or psychological deficits), and (8) upper good recovery (patient is able to resume normal life with the capacity to work without disabling neurological or psychological deficits).[18] Our primary outcome was recovery of consciousness at discharge, defined by eyes opening and the ability to follow commands. We selected re-emerging of consciousness as a primary outcome since it is an early stage of recovery preceding functional recovery and is a reasonable target to capture in a retrospective study. The ability to follow commands before discharge was obtained through the chart review of the neurologist, neurosurgeon, neurointensivist, and ICU nurses. It was defined as the ability to follow one-step commands (such as sticking the tongue out, showing two fingers, etc.).

**Resting-State EEG.** EEGs were obtained as a standard of care at our institution to exclude seizures in comatose patients. EEGs were obtained using a 10–20 system of electrode placement, using 16–19 EEG channels with adjustments for drains/wounds online referenced to Cz. EEGs were recorded using digital video EEG bedside monitoring (Xltek; Natus Medical, Oakville, ON, Canada; low-pass filter 70Hz, high-pass filter 0.1Hz, sampling rate up to 256-512Hz; impedances < 10kOhm). EEG recordings had short traces of data followed by long periods of no data across all channels due to recording or data export settings. These periods of missing data could not be recovered and therefore we defined a minimum of 10-minute of continuous resting-state data to include a patient in the analyses. Each of the 10-minute segments was visually assessed and segments with pervasive muscular, movement artifacts, and interictal/ictal activities were discarded.

EEG analyses were carried out in Python using the MNE-Python 0.24.1 and Nice 0.1 packages in custom scripts.[19,20] A 60Hz notch filter (one pass zero-phase filter with length 1,691 samples) followed by a high-pass filter above 0.5Hz and a low-pass filter below 40Hz (finite impulse response one pass zero-phase filter with length 1,691 samples) were applied, and the data were referenced to the average of all channels and split into 2-second epochs. Noisy channels or epochs were either interpolated automatically or rejected using Autoreject 0.4.0.[21] Finally, recordings with a higher sampling frequency were down sampled to 256Hz. A minimum of 10 continuous minutes of non-rejected EEG data was required which met the following requirements. For group analyses, features for one segment per patient were used. Segments selected had more than 200 clean epochs and less than 7 rejected channels, yielding 70 patients that had a good recovery and 37 that had a bad recovery. The number of non-rejected epochs (good = 271.5 + 29.6, bad = 279.8 + 22.5, U = 1094, p = .19) and rejected channels (good = 2.1 + 1.6, bad = 2.4 + 1.5, U = 1132, p = .28) did not differ across groups. Under these criteria, we constructed two datasets: one with 107 (80%) patients who met the EEG criteria (E) and another with 92 (69%) patients who met both the EEG and HEP criteria (EH). For subjects with more than one 10-minute trial of data, one was selected at random from among those with the best perceived data quality.

We calculated the following EEG measures based on prior literature on assessment of consciousness in the acute, and chronic states as well as the anesthesia literature[9,22–27]:

- Power Spectral Density (PSD) for frequencies from 1 to 30 Hz was calculated following the Welch method using a window length of 128 samples with 100 samples overlap and a nfft of 4096 samples. Normalized and non-normalized spectral data were computed in four different frequency bands: delta (1–4 Hz), theta (4–8 Hz), alpha (8–13 Hz), and beta (13–30 Hz). PSD is a commonly used quantitative measure in EEG studies calculated as the squared EEG amplitude in a specific frequency range.[22,23,28]
- Permutation Entropy (PE) is a measure of signal complexity, for which the EEG data are transformed into a symbolic representation, and the distribution of the obtained patterns is quantified for each channel giving a measure of how irregular the signal is. The transformation involves taking consecutive sub-vectors of length $n$ of the signal defined by parameter $\tau$ that determines the number of samples between elements resulting in a frequency-specific transformation. PE in the theta band ($\tau = 8$, $n = 3$) has proven informative for the classification of DoC patients and is the measure used in this work.[22,29]
- Weighted Symbolic-Mutual-Information (wSMI) is a measure of long-range connectivity that quantifies global information sharing by evaluating the nonrandom joint fluctuations between two EEG signals following the same symbolic transformation as for the PE.[25]
- Kolmogorov complexity (Kolcom) measures the complexity of the EEG signal by quantifying the compressibility of the signal in each channel. It has been used more recently in acute disorders of consciousness.[22,30]

Since changes in these EEG markers over time and sensors have proven useful for categorizing patients with DoC, we performed four types of dimensionality reduction for each marker.[20] These included calculating the average across trials and channels (mEmCh), the average across trials and the standard deviation across channels (mEsdCh), the standard deviation across trials and channels (sdEsdCh), and the standard deviation across trials and the average across channels (sdEmCh). This resulted in 44 total EEG features.

**Heart-Evoked Potentials (HEP) and Heart Rate Variability (HRV).** The standard of care EEG at our institution has at least one electrocardiogram (EKG) channel that is recorded and time-synched with the EEG data in the digital video EEG bedside monitoring (Xltek; Natus Medical).

For each recording, we visually inspected the EKG and selected the channel showing the clearest QRS complex. For each 10-minute recording, Neurokit 0.2.0 functions implemented in custom python scripts were used to remove slow drifts from the EKG signal (0.5Hz high-pass butterworth filter with order 5), to remove power line noise (signal was smoothed with a moving average kernel with the width of one period of 60Hz) and to automatically detect heartbeats.[31] A patient specific threshold was defined to reject wrongly detected heartbeats and finally a visual inspection was carried out to reject heartbeats missed by the chosen threshold. Recordings for which no clear QRS complexes were observed were not analyzed. Heart rate (HR) and HRV were calculated on those segments. The HR was computed as the inverse of the average difference between consecutive R peaks (RR intervals). HRV was measured as the root mean square of successive differences between RR intervals.[32]

To obtain HEPs, we extracted the − 200ms to 800ms EEG data relative to each R peak (corresponding to the QRS complex), linear detrended each epoch, automatically rejected noisy epochs and computed the averaged signal in each EEG electrode in 10-minute of good data. For HR, HRV, and HEP results; we performed a group analysis (classified by recovery of consciousness on discharge). For this, one segment per patient with more than 300 clean epochs and with less than 7 rejected channels was selected, resulting in 68 patients that had a good recovery and 32 with a bad recovery. The number of epochs (good = 695.9 + 160.7, bad = 737.0 + 173.8, U = 871, p = .25) and rejected channels (good = 1.1 + 1.1, bad = 1.2 + 1.3, U = 947, p = .55) did not differ across groups. We analyzed the data for 100 (75%) patients.

**Statistical Analysis.** Descriptive data were generated to describe patients who recovered consciousness versus patients who did not recover. Continuous and categorical variables were summarized using means and frequencies (%), respectively. Chi-square tests were used to examine relations between categorical variables. Mann-Whitney U tests were used to examine group differences for continuous variables, respectively. A value was considered an outlier and discarded if it was below or above 3 standard deviations.

We used the following machine learning models to predict recovery of consciousness on discharge: Random Forest with 500 decision trees (RF), Support Vector Machine with a linear kernel (SVM), Histogram-Based Gradient Boosting (HGB), and XGBoost (XGB). We calculated the models' area under the receiver operating characteristics curve (AUC-ROC). We also computed the reduction in the Residual Sum of Squares to report the most important variables contributing to our models. Class imbalance was accounted for using SMOTE with one neighbor.[33] We used stratified 10-fold cross-validation, where the models were trained on 90% of the samples and tested on the remaining 10%, repeated such that each sample appeared in the testing set once and the class balance was maintained in each fold. We repeated this process 100 times, yielding a series of 1000 AUC values, which we used to compare model performances. We trained models on basic clinical characteristics (age, motor response, and pupils' reactivity – the core IMPACT score) the Marshall CT score, HEP (Cz channel values) and rsEEG, as well as every combination of those features for both datasets.[5] Training samples were normalized using a standard scaler, which removed the need for non-normalized spectral power features. We performed manual feature reduction on the remaining 28 rsEEG features through an iterative test, concluding that mEmCh and mEsdCh features together yielded the highest AUCs. Therefore, for the remainder of this paper, rsEEG refers to the 14 mEmCh and mEsdCh features. All data analysis was conducted using the scikit-learn package in a custom Python script. Statistics on the HEP responses were done using a nonparametric cluster corrected permutation test for two-time windows (0 to 600ms and 600 to 800ms over all channels).[34]

**Data Availability.** The data supporting the findings of our work and the scripts written are available upon reasonable request.

## Results

**Subjects.** We analyzed the data for 113 (84%) patients who had a 10-minute segment of EEG data which met the requirements. Patients who recovered consciousness were younger (40 vs. 50, p = .01), less likely to have non-reactive pupils (12% vs. 35%, p = 0.0019). There were no differences between the two groups in sex, race/ethnicity, comorbidities, GCS on admission, CT Marshall Score, injury type, mechanism of injury, seizures, and sedation. Baseline and hospital characteristics are described in Table 1.

Table 1
Characteristics and Outcomes of TBI Patients who Recovered vs. Patients who did not Recover Consciousness Prior to Hospital Discharge

| **Characteristics/Outcome*+x** | **Recovery of Consciousness On Discharge n = 73 (64.6%)** | **No Recovery on Discharge n = 40 (35.4%)** | **P Values** |
|---|---|---|---|
| Age (SD), y | 40.4 (17.9) | 50.1 (20.4) | **.01** |
| Male (%) | 59 (80.8%) | 32 (80.0%) | .92 |
| Race/Ethnicity (%) | | | .08 |
| White | 56 (76.7%) | 25 (62.5%) | |
| Black | 16 (21.9%) | 11 (27.5%) | |
| Other | 1 (1.37%) | 4 (10.0%) | |
| Hispanic | 34 (46.6%) | 11 (27.5%) | .26 |
| Charlson Comorbidity Index (SD) | 0 (2) | 1.0 (1.7) | .26 |
| GCS (SD) | | | |
| Eye Response | 1 (1.0) | 1 (.30) | .73 |
| Motor Response | 4 (1.7) | 3 (1.6) | .49 |
| Verbal Response | 1 (0.96) | 1 (.52) | .14 |
| Pupils Reactivity (%) | | | **.0019** |
| Both Reactive | 61 (83.6%) | 21 (52.5%) | |
| One Reactive | 3 (4.1%) | 5 (12.5%) | |
| None-Reactive | 9 (12.3%) | 14 (35.0%) | |
| CT Marshall Score (%) | | | .26 |
| 1–2 | 41 (56.2%) | 16 (40.0%) | |
| 3–4 | 19 (26.0%) | 13 (32.5%) | |
| 5–6 | 12 (16.4%) | 10 (25.0%) | |

| Characteristics/Outcome[*+x] | Recovery of Consciousness<br><br>On Discharge n = 73 (64.6%) | No Recovery on Discharge n = 40 (35.4%) | P Values |
|---|---|---|---|
| Injury Type (%) | | | |
| SDH | 46 (63.0%) | 32 (80.0%) | **.05** |
| EDH | 8 (10.9%) | 3 (7.50%) | .56 |
| SAH | 56 (76.7%) | 30 (75%) | .81 |
| Hydrocephalus | 4 (1.70%) | 0 (0.0%) | .13 |
| Contusion | 45 (61.6%) | 25 (62.5%) | .87 |
| ICH | 8 (10.9%) | 8 (20.0%) | .21 |
| IVH | 19 (26.0%) | 12 (30.0%) | .62 |
| DAI on MRI | 13 (17.8%) | 4 (10.0%) | .21 |
| Mechanism of Injury (%) | | | .45 |
| Motor vehicle traffic accident | 27 (36.9%) | 11 (27.5%) | |
| Motor vehicle non-traffic accident | 5 (6.9%) | 7 (17.5%) | |
| Other road vehicle accident | 6 (8.2%) | 1 (2.5%) | |
| Water transport accident | 1 (1.4%) | 0 (0%) | |
| Vehicle accident not elsewhere classifiable | 2 (2.7%) | 3 (7.5%) | |
| Accidental fall | 22 (30.1%) | 12 (30.0%) | |
| Other accidents | 3 (4.1%) | 1 (2.5%) | |
| Suicide and self-inflicted injury | 1 (1.4%) | 0 (0%) | |
| Homicide or injury inflicted by others | 5 (6.9%) | 5 (12.5%) | |
| Injury undetermined whether accident/purposeful | 1 (1.4%) | 0 (0%) | |
| Penetrating Injuries (%) | 5 (6.9%) | 1 (2.5%) | .33 |
| Midline Shift (%) | 32 (43.8%) | 21 (52.5%) | .34 |
| Midline Shift Measurement (SD) | 7.3 (3.9) | 8.8 (4.9) | .24 |
| Seizure (%) | 6 (8.2%) | 10 (25.0%) | **.02** |
| EEG day post-injury (SD) | 10.6 (16.3) | 9.6 (11.6) | .75 |

| Characteristics/Outcome*+x | Recovery of Consciousness<br><br>On Discharge n = 73 (64.6%) | No Recovery on Discharge n = 40 (35.4%) | P Values |
|---|---|---|---|
| Sedation levels over the 2 previous half-lives prior to EEG monitoring | | | .42 |
| None | 37 (50.7%) | 22 (55.0%) | |
| Minimal | 10 (13.7%) | 8 (20.0%) | |
| Low | 10 (13.7%) | 6 (15.0%) | |
| Moderate | 16 (21.9%) | 4 (10.0%) | |
| Deep | 0 (0.0%) | 0 (0.0%) | |
| Surgical Treatment (%) | | | |
| Hemicraniectomy | 27 (36.9%) | 18 (45.0%) | .41 |
| SDH/EDH evacuation | 3 (4.11%) | 4 (10.0%) | .21 |
| EVD | 28 (38.4%) | 14 (35.0%) | .72 |
| Other | 5 (6.85%) | 6 (15.0%) | .16 |
| Tracheostomy (%) | 30 (41.1%) | 21 (52.5%) | .24 |
| PEG (%) | 29 (39.7%) | 19 (47.5%) | .42 |
| VPS (%) | 7 (9.59%) | 2 (5.0%) | .39 |
| Hospital Length of Stay (SD), days | 54.9 (57.5) | 48.5 (71.7) | .61 |
| ICU Length of Stay (SD), days | 43.2 (65.6) | 35.9 (37.9) | .52 |
| GOS-E on Discharge (SD) | 4 (2.2) | 2.0 (.88) | **< .0001** |
| Mortality (%) | 6 (8.2%) | 28 (70.0%) | **< .0001** |
| WLST | 3 (4.1%) | 13 (32.5%) | **< .0001** |
| Discharge Disposition (%) | | | **< .0001** |
| Home | 20 (27.4%) | 0 (0%) | |
| Rehabilitation | 31 (42.5%) | 2 (5.0%) | |
| Nursing Facility | 5 (6.8%) | 6 (15.0%) | |
| LTACH | 8 (10.9%) | 3 (7.5%) | |
| Other | 3 (4.1%) | 1 (2.5%) | |

| Characteristics/Outcome*+x | Recovery of Consciousness<br><br>On Discharge n = 73 (64.6%) | No Recovery on Discharge n = 40 (35.4%) | P Values |
|---|---|---|---|

**Abbreviations**: SDH, subdural hematoma; EDH, epidural hematoma; SAH, subarachnoid hemorrhage; ICH, intracerebral hemorrhage; IVH, intraventricular hemorrhage; DAI, diffuse axonal injury; PEG, percutaneous endoscopic gastrostomy; VPS, ventriculoperitoneal shunting; WLST, withdrawal of life-sustaining therapy; LTACH, long-term acute care hospital.

*Data are presented as number (%) unless otherwise specified.

+Percentages may not total 100 because of rounding xChi-square tests were used to examine relations between categorical variables. Mann-Whitney U tests were used to examine group differences for continuous variables, respectively.

**Physiologic Features (EEG and heart measures).** Each patient had at least one 10-min segment of EEG data for analysis. On average EEG was performed on day 6 post injury. A total of 21% of patients who didn't recover had seizures versus 11% in those who recovered (p = .2).

Patients who recovered consciousness had higher Kolcom (mEmCh: U = 1688, p = 0.010, mEsdCh: U = 953, p = 0.025), higher normalized beta power (mEmCh: U = 1652 p = 0.003), with higher variability across channels (mEsdCh: U = 1534, p = 0.034), and epochs (sdEmCh: U = 1711, p = 0.004), lower normalized delta power (mEmCh: U = 981, p = 0.04) and showed higher variability in wSMI values across time and channels (sdEmCh: U = 1636, p = .026, sdEsdCh: U = 1639, p = 0.024) than those who didn't recover. (Fig. 1) There was no difference between the two groups in the early HEP time window (0-600ms, all clusters with p > 0.42) nor for the later time window (600-800ms, all clusters with p > 0.11). HR (U = 974, p = 0.48) and HRV (U = 963, p = 0.77) did not differ between the two groups. (**Figure S2**)

**Final Models to Predict Recovery of Consciousness on Discharge**. We used Tukey's Range Test to identify the best-performing model type (SVM, RF, HGB, XGB) for each feature set using the distributions of 1000 AUCs generated by each model. We found that for 16 of the 18 feature sets (88.89%, all except CT and HEP from Dataset EH), the SVM yielded higher AUCs (p < 0.05, **Table S1**). Therefore, all results discussed in the remainder of this paper will be those produced by SVMs. To better understand how each feature contributed to the predictive accuracy of the models, we computed the explained variance ($R^2$) of each feature with respect to recovery of consciousness (**Figure S3**).

This study aimed to demonstrate that resting-state EEG data would improve the predictive power of machine learning models with respect to recovery of consciousness. Including rsEEG features with clinical features in SVM training sets yielded consistently higher predictive accuracy of recovery of consciousness (one-tailed t-tests, dataset E: Clinical + EEG (AUC = 0.69, 95% CI: 0.68–0.7) > Clinical (AUC = 0.66, 95% CI: 0.66–0.67), p < 0.001; Clinical + CT + EEG (AUC = 0.67, 95% CI: 0.66–0.68) > Clinical + CT (AUC = 0.64, 95% CI: 0.63–0.65), p < 0.001; CT + EEG (AUC = 0.67, 95% CI: 0.66–0.68) > CT (AUC = 0.56, 95% CI: 0.55–0.57), p < 0.001). It is worth noting that the models trained on only rsEEG features (AUC =

0.69, 95% CI: 0.68–0.69) produced similar AUCs as models trained on Clinical and rsEEG features (two-tailed t-test: $p > 0.05$). The inclusion of HEP features did not improve prediction under any circumstances (one-tailed t-tests, dataset EH: Clinical + HEP (AUC = 0.62, 95% CI: 0.61–0.63) > Clinical (AUC = 0.63, 95% CI: 0.62–0.64), CT + HEP (AUC = 0.58, 95% CI: 0.57–0.59) > CT (AUC = 0.60, 95% CI: 0.59–0.61), Clinical + CT + HEP (AUC = 0.59, 95% CI: 0.58–0.6) > Clinical + CT (AUC = 0.60, 95% CI: 0.59–0.61), Clinical + CT + EEG + HEP (AUC = 0.63, 95% CI: 0.62–0.64) > Clinical + CT + EEG (AUC = 0.65, 95% CI: 0.64–0.66), EEG + HEP (AUC = 0.64, 95% CI: 0.63–0.65) > EEG (AUC = 0.66, 95% CI: 0.65–0.67), all $p > 0.05$). The performance of all machine learning models is summarized in **Table S1**.

**Other Outcomes.** The group of patients who recovered consciousness had higher GOS-E on discharge (3 vs. 2, p .007), and lower mortality (22% vs. 46%, p .007) compared to those who didn't recover consciousness. There was no statistical difference between the two groups in hospital and ICU length of stay, surgical procedures performed, and discharge disposition. (Table 1)

## Discussion

Prognostication after TBI remains challenging. The current prognostication models to predict functional outcomes use age, basic neurological assessments, and radiographic findings as predictors.[5–7] The uncertainty about the potential awakening and meaningful recovery may influence the decisions of care and rehabilitation.[35] In this study, we used rsEEG measures to add the underlying neurophysiologic brain activity to the currently used prognostication measures. We found that rsEEG measures are different between patients who recovered consciousness prior to discharge and those who didn't. EEG measures improved prediction for recovery of consciousness before discharge beyond the basic demographic, clinical, and imaging data.

The majority of rsEEG studies acutely after injury had limited number of patients and were based on the spectral power of EEG.[23] EEG did not predict recovery as evaluated by the GCS score within 5 days of injury in a study of 103 patients.[36] In other smaller studies, a higher percentage of alpha variability was associated with better outcomes as evaluated by the Glasgow Outcome Scale (GOS) and GOSE at 30-day, 6-month, and 12-month follow-up.[37–40] In one study by Rae-Grant et al, rsEEG predicted outcomes (based on GOS) at 3-month better than other neurophysiologic measures but not better than GCS scores.[41] Delta coma was shown to predict unresponsive wakefulness state, and death at 12-month in 53 TBI patients.[42] Our study is unique as it addresses an important question regarding awakening before hospital discharge, an important outcome that may influence the decision regarding medical care and disposition. In our study of a relatively large sample of 113 patients, we utilized novel rsEEG measures to include not only spectral power, but signal complexity and a measure to quantify global information sharing. The results for spectral, connectivity and the complexity markers are in line with previous findings in consciousness research. Kolcom has shown to be a robust marker for the classification of minimally conscious (MCS) and unresponsive wakefulness state (UWS/VS) patients.[20,27] Higher complexity is correlated to higher states of consciousness, as observed in our cohort. In relation to the spectral bands analyzed, slow waves are reported to be negatively correlated to the conscious state, such that higher power in the delta band is present in UWS/VS patients compared to MCS and healthy individuals.[25,27] In addition, an overall shift in the aperiodic component of the power spectrum consistent with an increase in power for low frequencies and a decrease in power for higher frequencies has been reported during anesthesia induced loss of consciousness, as well as during the sleep cycle.[43,44] This is consistent with our findings, as patients who did not recover consciousness showed an increase in delta and decrease in beta normalized power. Furthermore, the dynamics for the spectral and information theory markers are consonant with previous investigations that show MCS and UWS/VS patients can be discriminated by MCS having greater temporal fluctuations in long range connectivity in the theta band, as well as for power in higher frequency bands (theta, alpha and beta). In our study, patients who recovered consciousness showed increased variability across the 10 minutes of data for wSMI, and power within the beta band compared to patients that did not recover consciousness. Overall, the ongoing brain activity for the patients with better outcome was more complex, richer in high frequencies and had greater temporal dynamics for connectivity in the theta band.

Using a supervised machine learning approach to prognosticate awakening, accuracy improved from 0.66 based on the core IMPACT score measures to 0.69 when adding rsEEG measures from 10-minute artifact-limited EEG data. The results can be explained by using multiple rsEEG measures related to complexity and information sharing, in addition to the large sample used in our cohort. More recently the CONNECT-ME study evaluated similar rsEEG measures (to those evaluated in this study) in a mixed cohort of 87 acute brain injury patients (25 with TBI). The authors reported an AUC of 0.68 in predicting consciousness levels at ICU discharge.[22]

Heart rate and heart rate variability are markers of autonomic dysfunction after TBI.[45] Raimondo et al. have shown a link in 127 patients (25 with acute and chronic TBI) between residual cognitive processing and the modulation of autonomic markers when they are triggered by auditory stimuli.[34] The authors showed a modulation of the cardiac cycle triggered by the auditory stimuli in minimally conscious state patients but not in unresponsive wakefulness state patients. In another study, Candia Riveira et al. showed that the HEP can be used to distinguish the state of consciousness of patients with chronic disorders of consciousness.[46] In our study, we found no difference between patients who recovered and those who did not recover consciousness. More importantly, the HEP responses did not improve the abilities of the models to predict awakening. This contrast with the previous results can be explained by the different population studied in this cohort (acute coma patients versus awake patients with subacute and chronic disorders of consciousness).

Looking at basic characteristics, patients who recovered were younger and less likely to have non-reactive pupils. Although the core IMPACT model is not built to prognosticate awakening, predicting awakening was low when using age, pupils’ reactivity, and motor function. This is consistent with using the IMPACT model to prognosticate outcomes in prior studies as the agreement between observed and predicted probabilities is poor, and mortality and unfavorable outcomes are generally lower than predicted.[5–7]

Our study has multiple limitations. First, the retrospective and single center nature of the study prevents the generalization of our findings without external validation. Second, the imbalance between the two groups may have limited the performance of our models. Third, the EEG indication was not part of a study protocol which may have led to a selection bias. Fourth, EEGs were obtained mostly once as a standard of care, limiting the evaluation of the dynamic EEG signal overtime. Finally, although awakening before discharge is an important outcome, this time frame is relatively short, and patients might have recovered consciousness after discharge. Nonetheless, the study presents a large sample of TBI patients to study rsEEG measures in predicting awakening. Future prospective multicenter studies are needed to validate our findings and to evaluate short and long-term outcomes beyond the recovery of consciousness.

## Conclusion

In this retrospective single center study, we found that rsEEG measures are different between patients who recovered consciousness prior to discharge and those who didn't. The EEG measures improved prediction for recovery of consciousness prior to discharge in comatose traumatic brain injury patients beyond the basic clinical and imaging data. Future studies are needed to validate our findings using other centers datasets and prospectively collected data.

## Declarations

### Transparency, Rigor, and Reproducibility Statement

The analysis plan was pre-determined but not registered for this study. This is a retrospective study to demonstrate the value of resting state EEG in determining prognosis after TBI and it was not powered to demonstrate differences. We used p-value <0.05 for statistical significance. Imaging/EEG acquisition and analyses were performed by team members blinded to relevant characteristics of the participants, and clinical outcomes were assessed by team members blinded to imaging/EEG results. All equipment and software used to perform imaging and preprocessing are widely available from commercial sources. The data and the codes supporting the findings of our work are available upon reasonable request. We followed STROBE guidelines for reporting observational studies.

### Acknowledgements

Thank you to the attending physicians, fellows, residents, nurses, and support staff of the Department of Neurology at the University of Miami Miller School of Medicine and Neuroscience Intensive Care Unit at Jackson Memorial Hospital for their support.

The manuscript complies with all instructions to authors.

All the authorship requirements have been met and the final manuscript was approved by all authors.

The manuscript has not been published elsewhere and is not under consideration by another journal.

We confirm the adherence to ethical guidelines and ethical approvals (IRB) was obtained for the study.

We confirm the use of STROBE checklist for our study.

The study was funded in part by the National Institutes of Health under Award Number K23NS126577.

**Author Contribution statement**

Study concept and design: Ayham Alkhachroum, Kristine O'Phelan, Jan Claassen, and Jacobo D. Sitt

Acquisition of Data: Berje Shammassian, Danielle Bass, Gabriela Aklepi, Esther Monexe, Pardis Ghamasaee, Evie Sobczak, Daniel Samano, Ana B. Saavedra

Analysis and interpretation of data: Ayham Alkhachroum, Emilia Flo, Brian Manolovitz, Jacobo D. Sitt

Drafting the manuscript: Ayham Alkhachroum

Critical revision of the manuscript for important intellectual content: Nina Massad, Mohan Kottapally, Amedeo Merenda, Joacir Graciolli Cordeiro, Jonathan Jagid, Andres Kanner, Tatjana Rundek, Kristine O'Phelan, Jan Claassen, and Jacobo D. Sitt

Statistical analysis: Ayham Alkhachroum, Brian Manolovitz and Emilia Flo

Study supervision: Ayham Alkhachroum, Kristine O'Phelan, Jan Claassen, and Jacobo D. Sitt

**Disclosures and conflict of interest**

EF, BM, BS, HC, DB, AS, EM, PG, ES, DS, AS, NM, MK, AM, JGC, JJ, AK, KO, and JS report no disclosures relevant to the manuscript.

AA is supported by an institutional KL2 Career Development Award from the Miami CTSI NCATS UL1TR002736 and by the National Institute of Neurological Disorders and Stroke of the National Institutes of Health under Award Number K23NS126577 and R21NS128326.

TR is funded by the Florida Department of Health for work on the Florida Stroke Registry and by the grants from National Institutes of Health (R01 MD012467, R01 NS029993, R01 NS040807, 1U24 NS107267), and the National Center for Advancing Translational Sciences (UL1 TR002736 and KL2 TR002737).

JC is supported by grant funding from the NIH R01 NS106014, R03 NS112760, R21 NS128326, and the DANA Foundation. JC is a minority shareholder at iCE Neurosystems.

## Figures

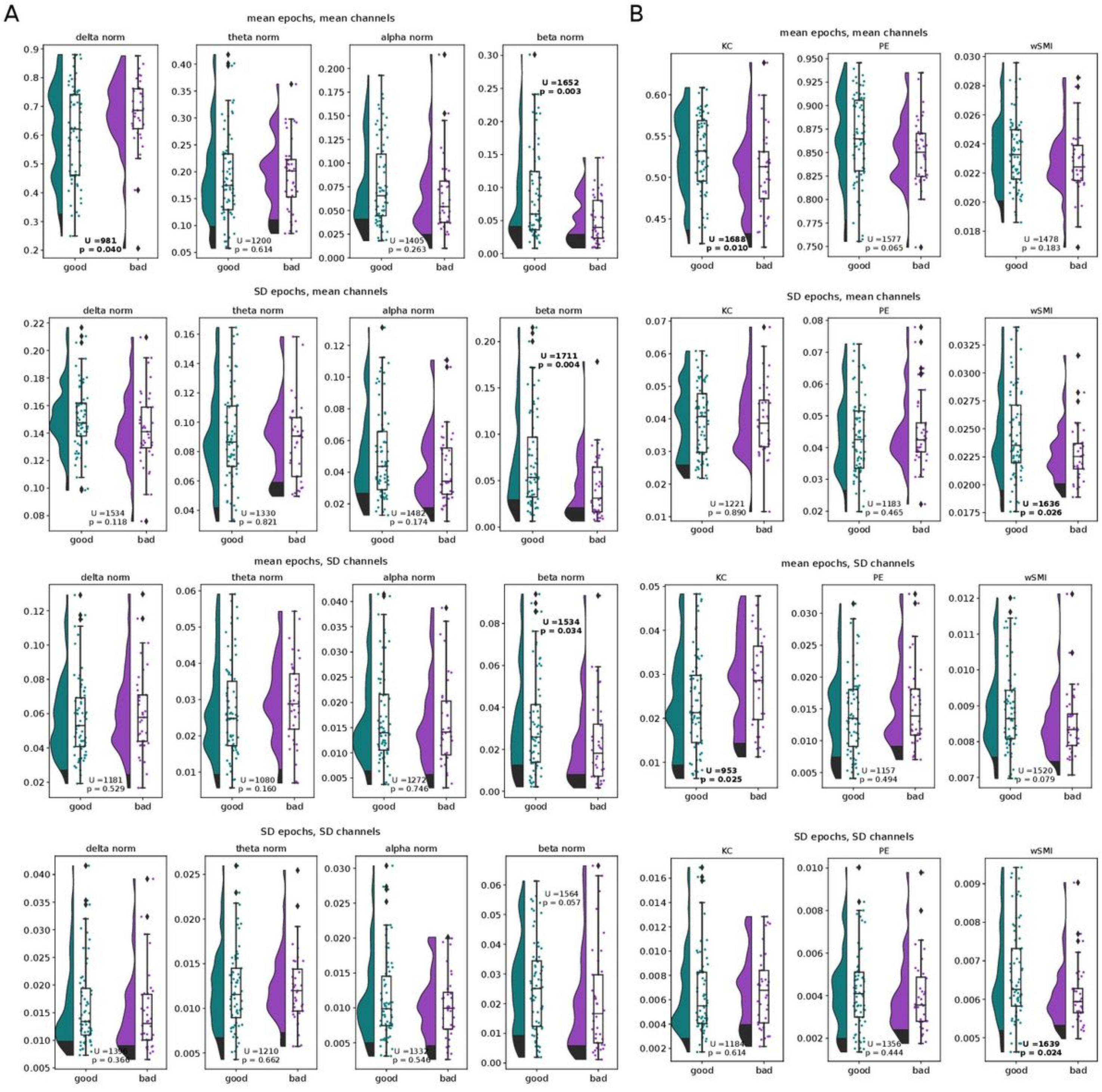

### Figure 1

Resting state EEG measures at a group level for patients who recovered and patients who did not recover consciousness prior to hospital discharge.

**Figure 1 caption** – Resting state EEG measures at a group level for patients who recovered (good) and patients who did not recover consciousness (bad) prior to hospital discharge. Resting measures include **A:** normalized power spectral density in different frequency bands (delta, theta, alpha, and beta), **B:** permutation entropy (PE), Weighted Symbolic-Mutual-Information (wSMI), and Kolmogorov complexity

(Kolcom). Each marker was evaluated for different dimensionality reductions of the data: the average across trials and channels (mean epochs, mean channels: mEmCh), the average across trials and the standard deviation across channels (mean epochs, SD channels: mEsdCh), the standard deviation across trials and the average across channels (SD epochs, mean channels: sdEmCh) and the standard deviation across trials and channels (SD epochs, SD channels: sdEsdCh). Patients who recovered consciousness had higher KC (mEmCh: U = 1688, p = 0.010, mEsdCh: U = 953, p = 0.025), higher normalized beta power (mEmCh: U = 1652 p = 0.003), with higher variability across channels (mEsdCh: U = 1534, p = 0.034), and epochs (sdEmCh: U =1711, p = 0.004), lower normalized delta power (mEmCh: U = 981, p = 0.04) and higher variability in wSMI values across time and channels (sdEmCh: U = 1636, p = .026, sdEsdCh: U=1639, p = 0.024) than those who didn't recover.

### Supplementary Files

This is a list of supplementary files associated with this preprint. Click to download.

- STROBEchecklist.pdf
- Supplemantary12324.docx
